# Chlorogenic acid alleviates the intestinal barrier dysfunction and intestinal microbiota disorder induced by cisplatin

**DOI:** 10.3389/fmicb.2025.1508891

**Published:** 2025-03-04

**Authors:** Ruiqi Tian, Yinchuan Ding, Shijie Zhang, Min Li, Yiran Wang, Qi Wu, Huanhuan Ding, Chengjie Song, Ce Shi, Min Xue

**Affiliations:** ^1^Department of Physiology, Xuzhou Medical University, Xuzhou, China; ^2^Department of Orthopedics, Nanjing Gulou Hospital Group Suqian Hospital, Suqian, China

**Keywords:** chlorogenic acid, cisplatin, intestinal microbiota, gut-barrier dysfunction, inflammation

## Abstract

**Introduction:**

The intestinal mucosal barrier is an important line of defense for the body, protecting it from intestinal bacteria, endotoxins, and antigens. Cisplatin, a clinical important chemotherapy medicine, is found the side effect with impairing intestinal epithelial cells’ structure and function, even causes intestinal mucositis which causes patients immense suffering and hinders the process of cancer treatment. Chlorogenic acid, as the component only second to caffeine in coffee, has been proved the contribution on cardiovascular and gastrointestinal benefits. So, we investigate the protective effect of chlorogenic acid on cisplatin induced intestinal barrier structure and function injury in mice from the perspective of gut microbiota.

**Methods:**

C57BL/6J mice were divided into 4 groups, including the control group, a cisplatin group, a chlorogenic acid treatment group receiving intraperitoneal injections alongside cisplatin (Cis + CGA1), and the last group pre-treated with chlorogenic acid before cisplatin administration (Cis + CGA2). The inflammation factor of IL-6, IL-1β, and TNF-α in colonic tissue and serum were detected, respectively. To explore the protection of chlorogenic acid on mucosal barrier’s integrity, we also detected the fecal LPS and the expression of occludin and ZO-1 proteins in colon tissue. And H&E staining was used to study the histopathological conditions of the colon tissue. Moreover, this article also utilized16S rDNA sequencing to analyze the gut microbiota of feces.

**Results:**

Chlorogenic acid administration reduced IL-6, IL-1β, and TNF-α level in both colon tissue and serum compared to the cisplatin alone treatment group. Furthermore, chlorogenic acid pretreatment notably improved intestinal barrier integrity by enhancing the expression of occludin and ZO-1 proteins in colon tissues. Moreover, 16S rDNA sequencing showed that compared with the control group, cisplatin group showed a reduced microbiota diversity, elevating abundance of Proteobacteria and pro-inflammatory environment of the increased Firmicutes/Bacteroidetes (F/B) ratio. However, chlorogenic acid treatment especially the pretreatment reversed the reduced microbiota diversity, elevating abundance of Proteobacteria and F/B ratio.

**Discussion:**

Microbiota diversity and all results suggest that chlorogenic acid treatment was able to mitigate these intestinal microbiota disorder and diversity reduction induced by cisplatin, effectively offer a protective effect against the inflammatory response and destruction of the mucosal barrier in the intestines caused by cisplatin.

## Graphical abstract

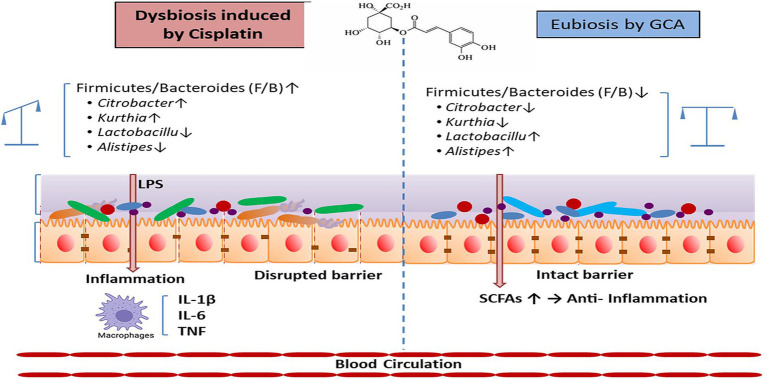

## 1 Background

As a broad-spectrum anticancer drug, cisplatin has remarkable clinical efficacy and low price and is an indispensable medicine in chemotherapy (Ali et al., 2022). Nevertheless, chemotherapy-induced enteric nervous system toxicity and gastrointestinal mucositis remains a dose-limiting condition impacting individuals undergoing chemotherapy (Greimel et al., 2006). So far, loss of mucosal integrity leading to intestinal barrier dysfunction is considered as one of the most prevalent complications among patients receiving chemotherapy (Sougiannis et al., 2021). Moreover, gut microbes disorder, which induces gut-barrier dysfunction, also leads to ulcers, pain and gastrointestinal bleeding, etc. (Wardill and Bowen, 2013). Currently, symptomatic mucositis treatment is mainly used, including mucosal protective agents, antibiotics, analgesics, local antibiotics, and cryotherapy, but the clinical effect could be more optimistic (Zhang et al., 2019).

Chlorogenic acid (CGA) belongs to the category of dietary phenols (Campa et al., 2003; Naveed et al., 2018; Miao and Xiang, 2020). Over the past few years, researchers have found that CGA, as the component only second to caffeine in coffee, is the main factor contributing to cardiovascular and cerebrovascular benefits (Lukitasari et al., 2020; Surma and Oparil, 2021; Huang et al., 2023). Besides that, CGA has shown a significant antioxidant and free radical scavenging effect. And it also plays anti-inflammatory, antiviral, and nerve protection functions (Tajik et al., 2017; Kumar et al., 2020). In this study, CGA was used to improving the intestinal mucosal injury induced by cisplatin in mice. Therefore, the purpose of this study was to investigate whether CGA restore cisplatin-induced intestinal mucosal structure and function injury in mice by modulating the gut microbiota and reducing colonic inflammation.

## 2 Materials and methods

### 2.1 Main instruments and reagents

Chlorogenic acid (P815485-500MG, Machlin Biochemical Technology Co., Ltd., Shanghai, China) was purchased from MACKLIN. For zonula occludens-1 (ZO-1) primary antibodies (ab221547, Cambridge, United Kingdom) was purchased from Abcam. Occludin primary antibodies (ab216327, Cambridge, United Kingdom) was purchased from Abcam. Beta Actin primary antibody (66009-1-Ig Wuhan, China) was purchased from Proteintech Company. Interleukin-1β (IL-1β) ELISA kit (JL18442, Shanghai, China), Interleukin-6 (IL-6) ELISA kit (JL20268, Shanghai, China), tumor necrosis factor-α (TNF-α) ELISA kit (JL10484, Shanghai, China) and lipopolysaccharides (LPS) ELISA kit (JL20691, Shanghai, China) were purchased from JonlnBio. Polyvinylidene difluoride (PVDF) (IPVH00010, Massachusetts, United States) was purchased from Millipore. RIPA lysis buffer (PC101, Shanghai, China) was purchased from EpiZyme. Protease Inhibitor Cocktail (HY-K0010, State of New Jersey, United States) was purchased from MedChemExpress. Primers were sourced from Sangon Biotech (Zhenjiang, China).

### 2.2 Experimental animals and groups

The experimental subjects of 6–8 weeks C57BL/6J male mice with a weight range of 20–25 g were obtained from the Experimental Animal Center of Xuzhou Medical University [License Number SYXH (Su) 2020-0048]. All the animals were acclimatized to the laboratory environment at room temperature for a week before the experiment complied with ethical principles and received approval from the Ethical Review Board of Xuzhou University of Chinese Medicine (Ethics Approval Number 220209S077).

**Figure 1:**
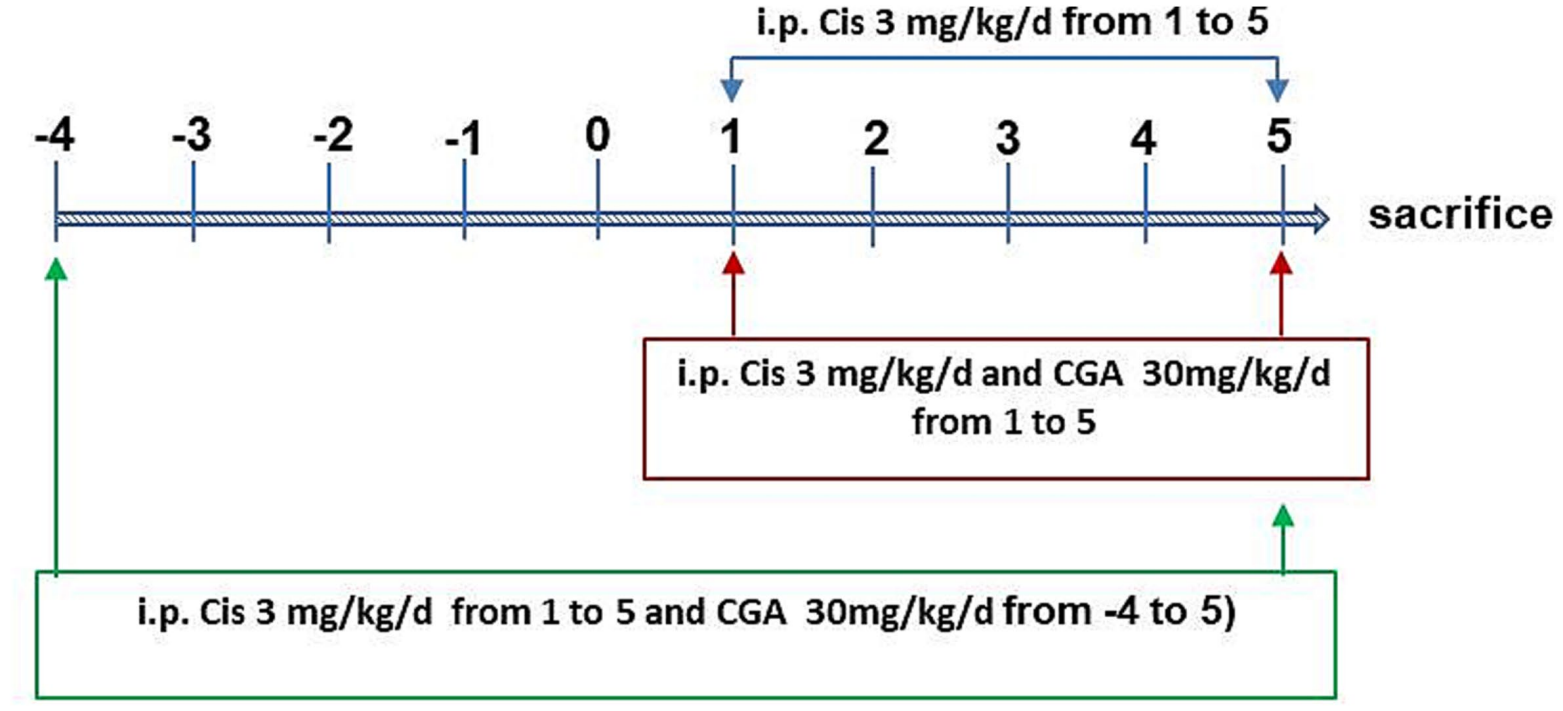
Experimental study design.

We randomly divided 28 mice into four groups of seven each. The control group, which served as the control, got an intraperitoneal injection of the same amount of physiological saline. The model group (cisplatin group) received intraperitoneal injection of cisplatin with 3 mg/kg (dissolved in 0. 9% saline solution) for total 5 days (Hardowar et al., 2024). The CGA conditioning group (Cis ± CGA1 group) was treated with cisplatin and CGA (intraperitoneal injection with 30 mg/kg/day, for total 5 days) at the same time. The CGA pretreatment group (Cis ± CGA2 group) was treated intraperitoneal injection with 30 mg/kg/day starting 5 days before cisplatin treatment and continuing throughout the whole experiment. Two hours after the 10th-day dose, the mice underwent euthanasia with 10% chloral hydrate intraperitoneal injection. As depicted in Figure 1, colon and serum samples were collected. Colon tissues were subsequently divided, with one portion fixed in 10% formalin for histological analysis and the other flash-frozen at −80°C for further investigations.

### 2.3 ELISA detection for serum IL-6, IL-1β, and TNF-α level

In order to detect the levels of inflammatory factors in serum and LPS content in mouse feces, enzyme linked immunosorbent assay (ELISA) method was used for detection. The collected mouse whole blood samples were left at room temperature for 2 h and then centrifuged at 1,000 × g for 20 min to remove the supernatant. The concentrations of inflammatory factors: IL-1β, IL-6, and TNF-α in the supernatants were then quantified using ELISA kits. A 20 mg of each fecal sample collected from the mice was subjected to a 50-min sonication on ice in 10 mL of sterile PBS to achieve homogenization. The supernatant was obtained and filter-sterilized after centrifuging it at 4,000 g for 10 min. The concentration of LPS in the purified supernatant was then quantified using an ELISA kit.

### 2.4 Quantitative real-time polymerase chain reaction assay

Total RNA was extracted from colon tissue using the Trizol method, followed by amplification of the target gene using polymerase chain reaction (PCR), which facilitated the detection of IL-6, IL-1β, and TNF-α mRNA expressions via real-time fluorescence quantitative PCR. The reaction conditions were denaturation at 95°C for 10 min, amplification reaction at 95°C for 15 s, 60°C for 60 s, 40 times, dissolution curve at 95°C for 15 s, 60°C for 60 s, 95°C for 15 s. After completing fragment amplification, calculate the results using the $2^{-\mathrm{\Delta \Delta CT}}$ method. Supplementary Table S1 shows the content related to specific primers.

### 2.5 Western blotting assay

Frozen colon tissues were cut into small pieces and lysed with RIPA lysis buffer (Shanghai, China). Phenylmethanesulfonyl fluoride (PMSF) was added (MedChemExpress, State of New Jersey, United States), and the tissue was homogenized using a tissue grinder (Wuhan Xavier Biotechnology Co., Ltd., Wuhan, China), followed by centrifugation at 12,000 rpm for 15 min at 4°C, and the supernatant was collected. Protein concentrations were quantified using BCA Protein Assay reagent (Proteintech, Wuhan, China). Equal amounts of protein samples were separated by electrophoresis on a standard Tris-glycine SDS-PAGE gel and then transferred to a polyvinylidene difluoride (PVDF) membrane. After blocking with 5% skim milk for 2 h at room temperature, PVDF membranes were incubated with primary antibodies overnight at 4°C. Primary antibody and dilution were as follows: ZO-1 (ab221547, Cambridge, United Kingdom) 1:1,000; occludin (ab216327, Cambridge, United Kingdom) diluted 1:1,000.

### 2.6 Immunohistochemistry

Colon tissue paraffin slices were washed with xylene for 15 min three times and dehydrated through gradient ethanol (such as 70, 80, 90, 95, 100% ethanol), and last washed with distilled water. Then the slices were blocked with 3% BSA (Bovine Serum Albumin) at room temperature for 30 min. Next, added primary antibody of ZO-1 (AF5145, Affinity, 1:100) and occludin (GB111401, Sayville, 1:400) for overnight incubation at 4°C. Dropwise added the secondary antibody (horseradish peroxidase labeled) to the slices corresponding to the species of the primary antibody and cover the tissue for incubation at room temperature for 50 min. Last, fresh DAB solution were used for coloration and positive staining appeared as brownish-yellow.

### 2.7 16S rDNA high throughput sequencing

#### 2.7.1 DNA extraction and PCR amplification

This article chose to use the Mag Bind fecal genomic DNA kit when extracting genomic DNA from samples, and strictly followed the instructions of the kit during the extraction process. After the extraction operation was completed, the quality and concentration of DNA were detected by agarose gel electrophoresis and NanoDrop^®^ ND-2000 spectrophotometer. In order to conduct further research and analysis, the target fragment was placed in an environment of −80°C. The amplification operation was performed on the V3-V4 highly variable region of the 16S rRNA gene in bacteria, using primers 338F (5′-ACTCCTACGGGGGGCAG-3′) and 806R (5′-GACTACHVGGGTWTCTAAT-3′). Utilizing ABI GeneAmp^®^ The 9700 thermal cycler was used for amplification, and the reaction system prepared in this article had a volume of 20 μL. The DNA template was 10 ng, including FastPfu polymerase and 5 μM primers, 2.5 mM dNTP, 5× FastPfu buffer, etc. Set the reaction time to 95°C for 3 min, followed by 95°C for 30, with 27 cycles, followed by 55°C, 30 s, 72°C, 45 s, and finally 72°C for 20 min. In order to make the research results of this article more realistic and accurate, three samples were amplified for each sample. To purify the target product, agarose gel unit is used, and Quantus^™^ Fluorescence meter is used for quantitative analysis of the product.

#### 2.7.2 Illumina sequencing

According to the guidelines of Majorbio Bio Pharm Technology Co., Ltd., pairwise end sequencing was performed on the Illumina PE300/PE250 platform using purified amplicons with equal molar weight combinations.

#### 2.7.3 Data processing and statistical analysis

De-multiplexing of the raw FASTQ files was performed using an in-house Perl script. Then, we applied FLASH version 1.2.11 and FASTQ version 0.19.6 (Chen et al., 2018; Magoc and Salzberg, 2011) filter and merge the reads based on these criteria. A strategy of exact barcode matching was utilized, permitting up to a two-nucleotide mismatch in the primer sequences. After completing the optimization operation, use UPARSE 11 (Edgar, 2013) to perform sequence clustering, making it a classification unit (OTU), and set the sequence similarity threshold to 97%. The most frequently occurring sequence within each OTU was designated as its representative. Subsequently, chloroplast sequences were manually removed from the OTU table. Rarefied the 16S rRNA gene sequence in each sample to minimize the impact of sequencing depth changes on the diversity analysis of results. The average Good’s coverage was still 99.09% even after rarefaction.

Finally, we used RDP Classifier to assign the classification method to the OTU representative sequences in the gene database, and clarified the confidence threshold to 0.7. Using PICRUSt2 (Douglas et al., 2020), the functional potential of the microbial communities was subsequently inferred, which analyzed the representative sequences.

#### 2.7.4 Statistical analysis

Our study analyzed the gut microbiota of bioinformatics using the Majorbio Cloud platform. When calculating the alpha diversity index, Mothur v1.30.2 was chosen to detect content such as Chao1 richness and OTU. Sparse curves were also plotted to evaluate the adequacy of the use. Implement principal coordinate analysis (PCoA) using Vegan v2.4.3 software. The PERMANOVA test, via the same Vegan package, determined the extent of variation due to treatment and its statistical significance. When studying and analyzing the effect size (LEfSe), linear discriminant analysis (LDA) was chosen to identify significantly enriched taxonomic groups in the population. The LDA score threshold was greater than 2, and the *p*-value was less than 0.05. When conducting research and analysis on the relationship between gut bacterial community structure and cisplatin, distance based redundancy analysis (db-RDA) was chosen. Finally, linear regression analysis was chosen to study the relationship between microbial diversity index and platinum induced significant changes.

### 2.8 Statistical analyses

Data presented in this study are the averages of three independent experiments. Statistical analyses were conducted using SPSS 20.0 and GraphPad Prism 7.0 software. Statistical significance was defined based on standard deviation. Student’s *t*-test or one-way analysis were used for statistical analyses between two or multiple groups. Statistical significance was set at *p* < 0.05.

## 3 Results

### 3.1 CGA reduced the levels of inflammatory factors in the serum

**Figure 2:**
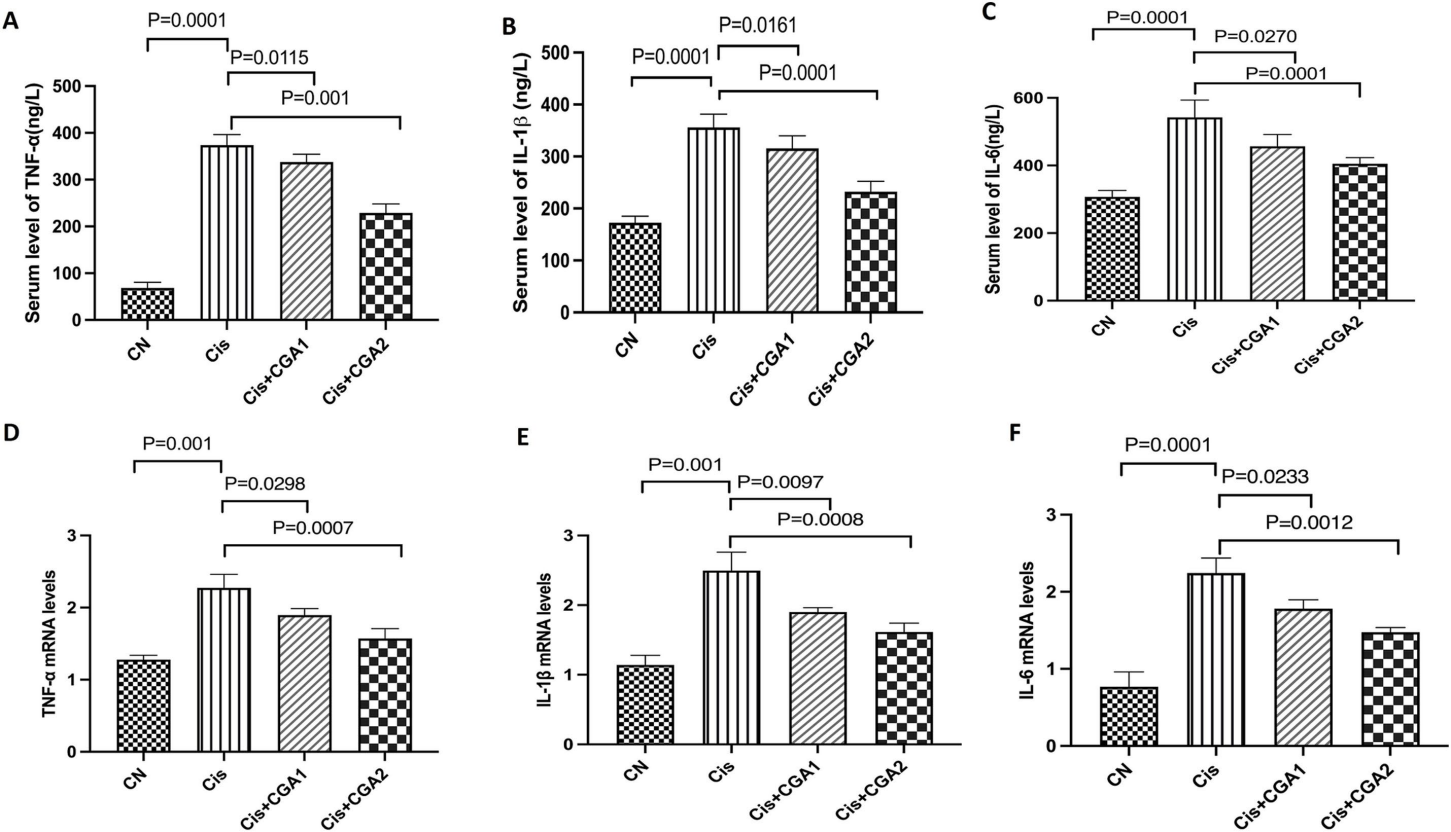
The effect of CGA on the secretion levels of the immune cytokines in mouse colon and in serum. **(A–C)** IL-1β, IL-6, and TNF-α levels in serum. **(D–F)** IL-1β, IL-6, and TNF-α mRNA expression in colon.

When testing the levels of various inflammatory factors in serum, ELISA was chosen. Comparing the control groups, it can be clearly seen that the cisplatin group has significantly higher levels of inflammatory factors: TNF-α, IL-1β, and IL-6 (*p* = 0.0001, *p* = 0.0001, *p* = 0.0001). This article also chose to use RT qPCR to detect the expression levels of inflammatory factors of colon in different groups. The results showed that compared with the control group, in the cisplatin group, the mRNA levels of TNF-α, IL-1β, and IL-6 were significantly increased (*p* = 0.0001, *p* = 0.0001, *p* = 0.001), and CGA pretreatment significantly reduced the levels of inflammatory factors both in the serum (*p* = 0.001, *p* = 0.001, *p* = 0.001) and colon (*p* = 0.0007, *p* = 0.0008, *p* = 0.0012) (Figure 2).

### 3.2 CGA protected the mucosal barrier by modulating occluding and ZO-1 protein expression

**Figure 3:**
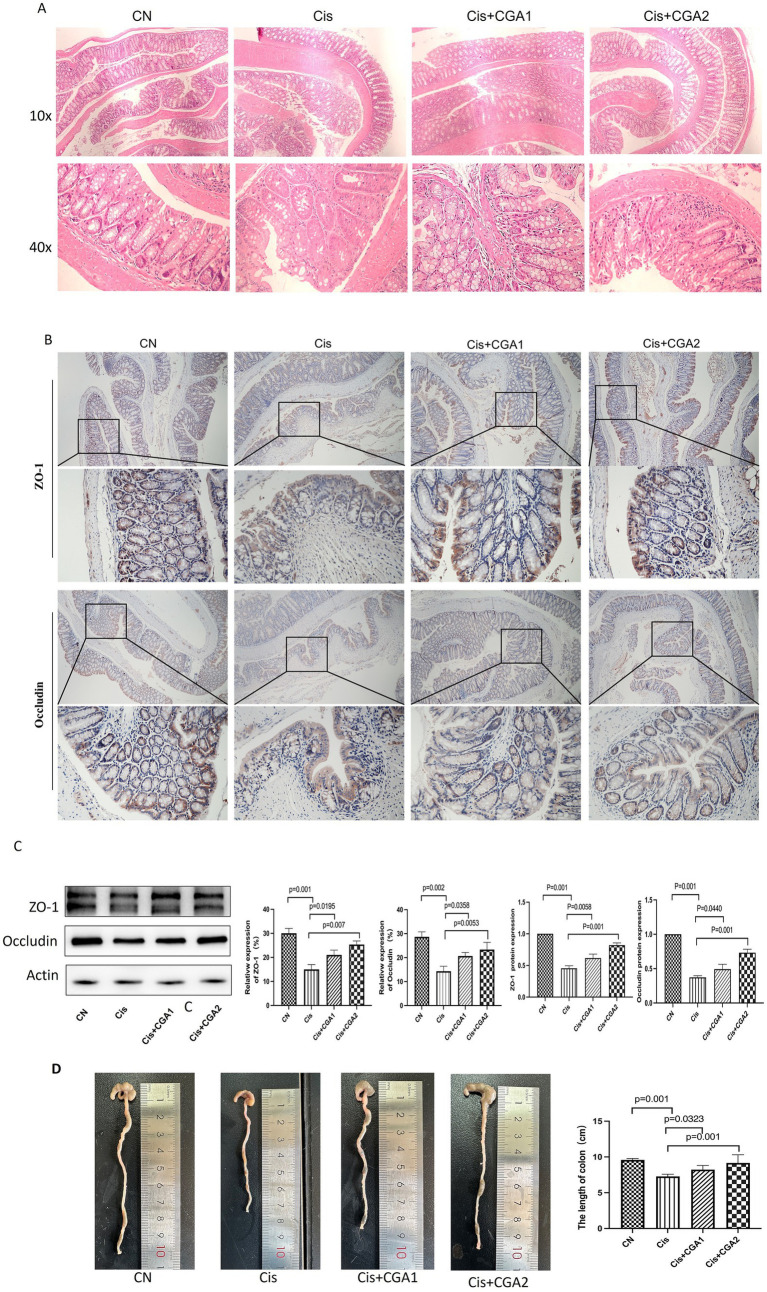
The effect of CGA on the mouse colon injury induced by cisplatin. **(A)** The optical microscopy observation of the colon tissue after HE staining. **(B)** ZO-1 and occludin immunohistochemical staining of the colon tissue. **(C)** ZO-1 and occludin proteins relatively expression in different groups. **(D)** Colon length of mice in different groups.

As Figure 3A showed the colon tissue in the control group revealed a well-defined serosal membrane, muscular layer, submucosa with neatly arranged glands. In the cisplatin model group, the structure was disorganized with obviously destroyed glands and a large number of inflammatory cells infiltration. CGA especially pretreated mice had intact intestinal mucosa, regular glandular structure. Figure 3C show that comparing the cisplatin model group with the control group, it can be concluded that the former had significantly lower levels of ZO-1 protein expression and occludin protein expression compared to the latter (*p* = 0.001, *p* = 0.002); After receiving CGA pretreatment, it can be clearly observed that the expression levels of these two proteins had significantly increased compared to the model group (*p* = 0.007, *p* = 0.0053). Immunohistochemical results (Figure 3B), showed that compared with control group, the expression of ZO-1 protein (*p* = 0.001) and occludin protein (*p* = 0.001) in cisplatin group was significantly decreased. After CGA treatment, especially pretreatment, the expression of ZO-1 and occludin protein was increased (*p* = 0.001, *p* = 0.001).

Colonic shortening resulting from histological alterations triggered by cisplatin stimulation and is commonly utilized as a morphological marker for intestinal inflammation (Shao et al., 2020). Comparing the cisplatin and control groups, it can be clearly seen that the former had a shorter colon compared to the latter (*p* = 0.001) (Figure 3F), indicating cisplatin can cause colon damage in mice. The damage was significantly improved after treatment with CGA, especially in CGA pretreatment group (*p* = 0.001). These results indicate that CGA does have a protective effect on the colon injury caused by cisplatin.

### 3.3 Analysis of 16S rDNA sequencing results of fecal contents in each group

#### 3.3.1 CGA restored the microbial abundance and microbial community diversity

**Figure 4:**
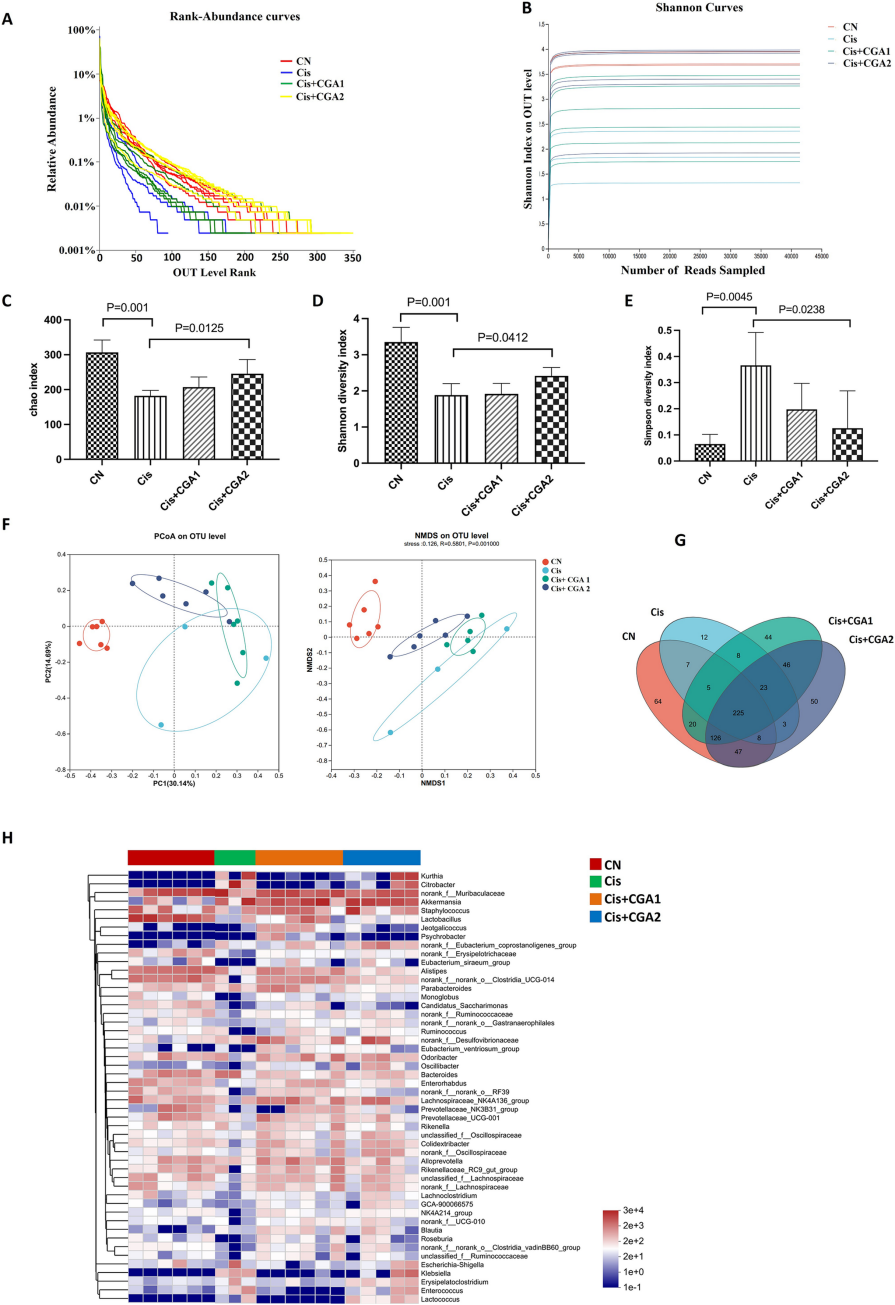
Analysis of 16S rDNA sequencing results of fecal contents in each group. **(A)** Rank-abundance curves. **(B)** Shannon curve. **(C-E)** Index of Chao1, Shannon and Simpson. **(F)** Beta diversity analysis of PCoA and NMDS. **(G)** Venn diagram. **(H)** Community heatmap analysis on genus level.

The dilution curve exhibited a tendency to flatten, indicating that sequencing depth had largely reached saturation, and the amount of sequencing data obtained was deemed reasonable. During the four groups, the curve of cisplatin group was the lowest, indicating fewer species and lower species diversity in cisplatin group, as shown in Figures 4A,B. Comparing the control group with the cisplatin group, it was clear that the microbial diversity level of the cisplatin group was significantly lower than that of the control group with a decreased Chao index (*p* = 0.001) and Shannon index (*p* = 0.001) and an increased Simpson index (*p* = 0.0045). On the contrary, CGA pretreatment recover the reduced species diversity induced by cisplatin with upregulating the Chao index (*p* = 0.0125) and Shannon index (*p* = 0.0412) and downregulated Simpson index (*p* = 0.00238) (Figures 4C–E). Through in-depth research and analysis, it can be clarified that there were differences in the richness and diversity of biological communities among different populations. The analysis methods such as pan/core species curves emphasize the changes in community structure and composition of different species. The research results of this article can quantitatively measure the relationship between microbial community composition and diversity and CGA and cisplatin, clarifying the important role that microbial community dynamics can play in cisplatin induced intestinal injury (Figures 4F–H).

#### 3.3.2 CGA improved gut microbiota disturbance induced by cisplatin

**Figure 5:**
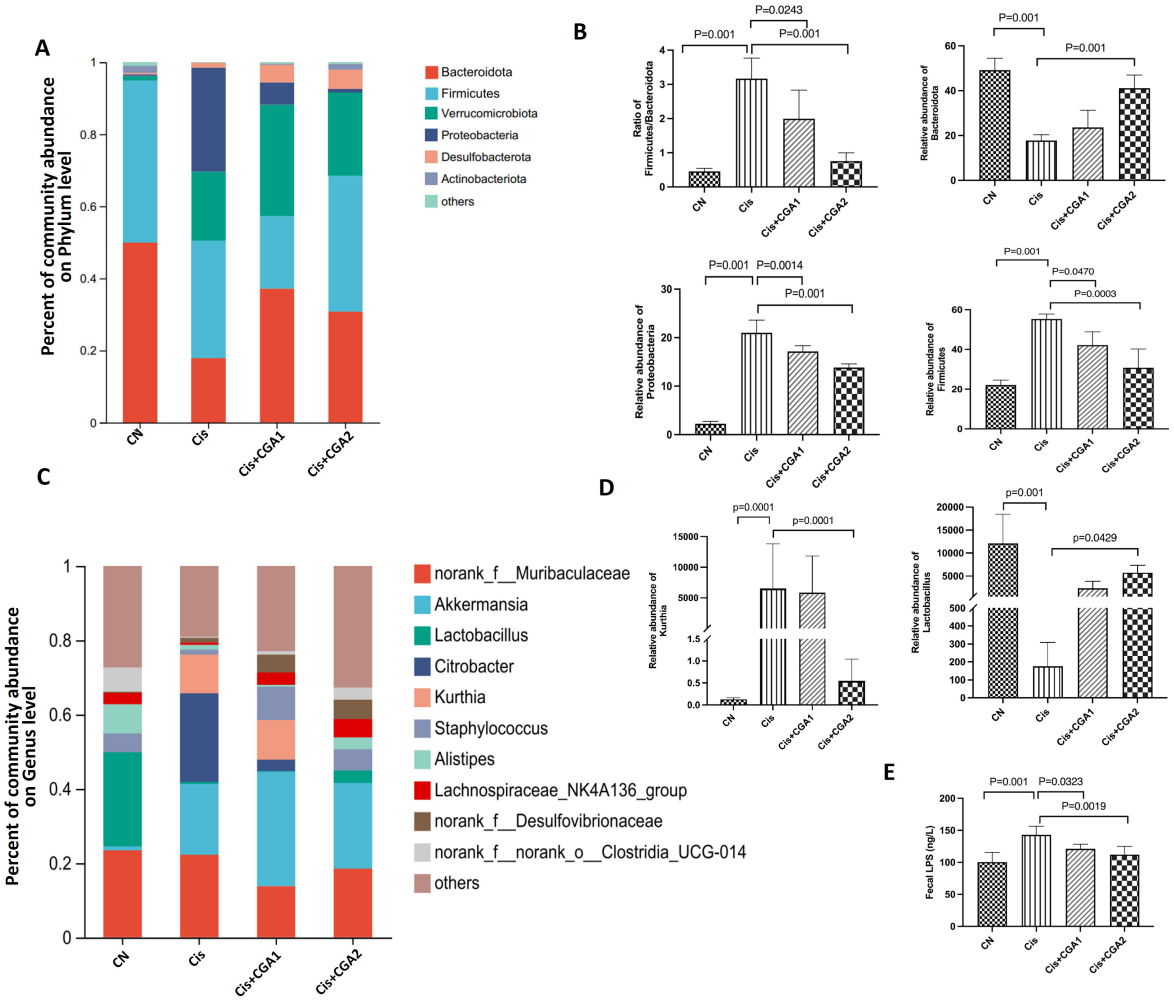
Intestinal microbiota composition analyses. **(A–D)** Percent of community abundance on phylum level. **(E–G)** Relative abundance of on genus level of different groups. **(H)** Fecal LPS level of mice in different groups.

The results of species difference analysis indicate that the absolute dominant species of control group mice are Bacteroidetes and Firmicutes. In comparison, Proteobacteria and Verrcomicrobiota are the top 2 Phylum in cisplatin group (Figure 5A). Besides that, as the Firmicutes and Bacteroidetes ratio was significantly higher in the cisplatin group compared with the control group (*p* = 0.001). However, CGA pretreatment could reverse the proportion (*p* = 0.0243). Compared with control group, relative abundance of Bacteroidetes decreased significantly in cisplatin treatment group (*p* = 0.001), while the relative abundance of Proteobacteria increased greatly. And CGA pretreatment could reverse the above phenomenon induced by cisplatin (Figures 5B,C). We also compared the ratio of Firmicutes/Bacteroidetes in different groups. There is an increased ratio in cisplatin treatment (*p* = 0.001) and CGA pretreatment reversed the increased ratio induced by cisplatin (*p* = 0.0243) (Figure 5D). At the genus level, compared with the control group, the relative abundances of Lactobacillus in cisplatin reduced significantly (*p* = 0.001). After CGA pretreatment, the abundance of *Lactobacillus* was significantly increased (*p* = 0.025). In addition, cisplatin also significantly increased the abundance of substances such as *Citrobacter* (*p* = 0.001), but CGA pretreatment could reduce the elevated abundance of *Citrobacter* induced by cisplatin (*p* = 0.001) (Figures 5E–G). This is supported by the relative abundance fluctuation of Lactobacillus which linked to intestinal barrier function and LPS production (Wu et al., 2021; Everard et al., 2013; Depommier et al., 2019). Similarly, the LPS content (Figure 5H) in the feces of the mice measured by ELISA showed that compared with the control group, the cisplatin group had a significant increase in LPS content (*p* = 0.001) and the CGA pretreatment group had a decrease in LPS content (*p* = 0.0019).

## 4 Discussion

The colon epithelial barrier primarily consists of monolayered intestinal epithelial cells and intercellular tight junction proteins which are crucial for preserving intestinal epithelial cells’ continuity and structural integrity. Dysfunction in this barrier contributes to increase intestinal mucosal permeability, thereby mediates the onset and progression of ulcerative colitis (Kumar et al., 2021). Two tight junctions composed of ZO-1 and occludin proteins (Jiang et al., 2021; Chu et al., 2021; Su et al., 2021) form the basis of the integrity of tight junctions. Occludin has the functions of sealing cell space, regulating paracellular transport and regulating signal conduction (Schulzke et al., 2005), etc., while ZO-1 affects cell proliferation, differentiation and growth, and regulates intercellular signal transduction and penetration (Guo et al., 2022). Cisplatin is found to impair intestinal mucosal barrier’s integrity by reducing the proteins expression both occludin and ZO-1. However, CGA pretreatment restored the injury of intestinal epithelium induced by cisplatin. Histopathological analysis using H&E staining of colon sections also validated these findings. The cisplatin-treated group had evident destruction of the epithelial structure, with damage to crypts and goblet cells. However, the control group and CGA pretreated group maintained the structural integrity of the colonic epithelium, with intact crypts and goblet cells, and minimal inflammatory cell presence.

The mRNA level of inflammatory factors in colon was further detected. It was found that the cisplatin group had significantly higher levels of inflammatory factors including IL-1β, IL-6, and TNF-α compared to the control group. Notably, pre-administration of CGA mitigated the increased inflammatory factors effectively. These outcomes suggest that CGA can partially ameliorate attenuate local and systemic inflammation. Besides that, colon shortening lead to nutritional deficiencies, electrolyte imbalances, and further impairments in the patient’s recovery and quality of life. And our experiment showed CGA pretreatment recover the length of colon with benefits for mice.

An increasing amount of evidence from extensive research confirms a close relationship between abnormal intestinal mucosal barrier function and disruption of gut microbiota (Guo et al., 2022; Zhou et al., 2023; Xu et al., 2022). The imbalance of microbiota can cause local and systemic immune responses in the gut, further promoting the occurrence and progression of local inflammation (Wang et al., 2024). Compared with the control group, there was a significant change in microbial diversity in the cisplatin group. The Simpson index showed an upward trend, while the Chao index and Shannon index showed a downward trend in cisplatin group. However, comparing the CGA pre-treatment group and the cisplatin group, it can be clearly seen that the former had a significantly improved Shannon index and Chao index. The results were considered that the relative abundance of cisplatin group’s microbial community had decreased at the phylum level, pretreatment with CGA can restore the depressed microbial diversity induced by cisplatin. Furthermore, the control group revealed the dominant phyla with Bacteroidetes and Firmicutes, whereas the dominant phyla in cisplatin administration group were Firmicutes and Proteobacteria. Phylum Firmicutes are bacteria with high peptidoglycan content in the cell wall and studies have shown that increased abundance with Firmicutes in the intestine can lead to more efficient absorption of food heat and thus lead to obesity (Wang et al., 2024). Usually, the indicator of Firmicutes/Bacteroidetes (F/B) can be used to evaluate the pro-inflammatory environment of the intestine (Ozsoy et al., 2022). Promoted F/B ratio indicates a triggering inflammation and related immune reactions (Salvi and Cowles, 2021; Allam-Ndoul et al., 2020). The results demonstrated that CGA pretreatment significantly improved the increased F/B ratio induced by cisplatin.

*Citrobacter* is a gram-negative bacterium that is part of the normal intestinal flora and also an important conditional pathogen. It is considered as a commensal bacteria and an opportunistic pathogen depending on the clinical picture (Tamura et al., 2003; El Qaidi et al., 2019). The research results showed an increased abundance of *Citrobacter* and reduced level of *Lactobacillus* in the cisplatin group on the genus. However, CGA pretreatment can confine the abundance of *Citrobacter* and partially restore the abundance of *Lactobacillus*. Besides that, the pretreatment with CGA also upregulated the abundance of *Akkermansia*. Despite a short history since its first isolation, *Akkermansia muciniphila* has been extensively studied in relation to its effects on human metabolism. A recent human intervention study also demonstrated that the bacterium is safe to use for therapeutic purposes. The best-known effects of *A. muciniphila* in human health and disease relate to its ability to strengthen gut integrity, modulate insulin resistance, and protect the host from metabolic inflammation.

All the above results indicate that CGA pretreatment can repair and position the junctions of epithelial cells, significantly reducing the damage caused by cisplatin. The principle of this process may be that CGA can regulate the disruption of bacterial communities induced by cisplatin, by regulating community richness and diversity to maintain a stable microenvironment, so as to prevent and alleviate the damage of intestinal health caused by cisplatin in mice and inhibit local and systemic inflammatory responses.

## Data Availability

The original contributions presented in the study are publicly available. This data can be found here: https://www.ncbi.nlm.nih.gov/, accession number PRJNA1229452.
