# Supplementary material for: Chlorogenic acid alleviates the intestinal barrier dysfunction and intestinal microbiota disorder induced by cisplatin
Source: Front Microbiol. 2025 Mar 4;16:1508891. doi: 10.3389/fmicb.2025.1508891 (PMC11919278; doi:10.3389/fmicb.2025.1508891)
Supplement: Supplementary file 1 [file Table_1.docx]

Supplementary table S1

Table 1 Primers sequence

|  | F： | R： |
| --- | --- | --- |
| IL-1β | ACTCACTTAAAGCCCGCCTG | TCAGAATGTGGGAGCGAATG |
| IL-6 | CGGGAACGAAAGAGAAGCTCTA | GAGCAGCCCCAGGGAGAA |
| TNF-ɑ | TCTTCTCGAACCCCGAGTGA | CCTCTGATGGCACCACCAG |
| β-actin | TGAGAGGGAAATCGTGCGTGAC | GCTCGTTGCCAATAGTGATGACC |
